# CD155 Promotes the Progression of Cervical Cancer Cells Through AKT/mTOR and NF-κB Pathways

**DOI:** 10.3389/fonc.2021.655302

**Published:** 2021-06-07

**Authors:** Lu Liu, Ying Wang, Chen Geng, Aihong Wang, Sai Han, Xuewu You, Yu Sun, Junhua Zhang, Wei Lu, Youzhong Zhang

**Affiliations:** ^1^ Department of Obstetrics and Gynecology, Qilu Hospital, Cheeloo College of Medicine, Shandong University, Jinan, China; ^2^ Key Laboratory of Gynecologic Oncology of Shandong Province, Jinan, China; ^3^ Shandong Engineering Laboratory for Urogynecology, Qilu Hospital of Shandong University, Jinan, China; ^4^ Department of Obstetrics and Gynecology, Yidu Central Hospital of Weifang, Weifang, China; ^5^ Department of Obstetrics and Gynaecology, Feicheng Hospital Affiliated to Shandong First Medical University, Tai’an, China

**Keywords:** CD155, cervical cancer, carcinogenesis, AKT/mTOR, NF-κB

## Abstract

Expression of the immunoglobulin superfamily member CD155 was increased in a variety of human malignancies, but the role of CD155 in tumorigenesis and tumor development in cervical cancer has not been elucidated. In this study, immunohistochemistry and enzyme-linked immunosorbent assay analyses showed that CD155 expression gradually increases with the degree of cervical lesions. In vitro and *in vivo*, reducing the expression of CD155 inhibited cell proliferation, cell viability and tumor formation and arrested the cell cycle in G0/G1 phase. Antibody array-based profiling of protein phosphorylation revealed that CD155 knockdown can inhibited the AKT/mTOR/NF-κB pathway and activated autophagy and apoptosis; the opposite effects were observed upon CD155 has overexpression. We proved that there is an interaction between CD155 and AKT by immunoprecipitation. We further confirmed the mechanism between CD155 and AKT/mTOR/NF-κB through rescue experiments. AKT knockdown reversed the anti-apoptotic effects and activation of the AKT/mTOR/NF-κB pathway induced by CD155 overexpression. Our research demonstrated that CD155 can interact with AKT to form a complex, activates the AKT/mTOR/NF-κB pathway and inhibit autophagy and apoptosis. Thus, CD155 is a potential screening and therapeutic biomarker for cervical cancer.

## Introduction

Cervical cancer is the fourth most common female malignancy in morbidity and mortality (1). Globally, there were more than 569,000 new cases of cervical cancer and 311,000 deaths in 2018 (1). The main cause of cervical cancer is human papillomavirus (HPV) infection (2). However, not all women infected with HPV develop cervical cancer, which may be due to differences in epigenetic modifications (3). The identification of new markers that can predict cervical cancer would aid diagnosis and potentially provide targets for treatment. One potential marker is CD155, also called NECL-5, a member of the immunoglobulin superfamily (4). CD155 has four splicing subtypes: α, β, δ, and γ. The α isoform contains an immunoreceptor tyrosine-based inhibitory motif (ITIM), which is essential for the effects of this subtype on tumor biology (5). CD155 regulates signal transduction (6), cell adhesion (7), motility (8), proliferation (9) and survival through the recruitment of tyrosine phosphatase 2 (SHP-2) *via* its ITIM. CD155 is overexpressed in many cancers, including lung adenocarcinoma (10), pancreatic cancer (11), ovarian cancer (12), myeloid leukemia (13), neuroblastoma (13, 14), colorectal cancer (14), and cholangiocarcinoma (15). However, potential role of CD155 in the development of cervical cancer is unknown.

In the present study, we detected CD155 expression in serum and tissue specimens from patients with cervical cancer or high-grade squamous intraepithelial lesions (HSIL) patients. We found that CD155 expression is associated with poorly differentiated cervical cancer. In addition, we knocked down and overexpressed CD155 *in vivo* and *in vitro* and assessed changes in cell function. Co-immunoprecipitation (Co-IP) assay and rescue assay confirmed that CD155 can interact with AKT to form CD155/AKT complex, and further promote the proliferation of cervical cancer cells and inhibit autophagy through AKT/mTOR and NF-κB pathways.

## Materials and Methods

### Human Tissue Samples

A total of 65 serum samples were collected, including 30 from cervical cancer patients, 20 HSIL patients, and 15 from subjects with normal cervix. In addition, we collected 100 paraffin-embedded tissue samples, including 66 cervical cancer samples, 16 HSIL samples, and 18 normal cervix samples. All samples were originally collected at Qilu Hospital of Shandong University. All patients were clinically staged according to FIGO guidelines. Clinical data were collected from the medical record management system of Qilu Hospital. The Ethics Committee of Qilu Hospital approved this study.

### Immunohistochemistry

Immunohistochemistry (IHC) was performed using the SP-9001 immunohistochemistry detection kit (Zhongshan Golden Bridge, Beijing, China) according to the manufacturer’s instructions. Each 4-μm-thick paraffin slice was deparaffinized and incubated with a CD155 antibody at 4°C overnight, followed by incubation with a biotin-labeled goat anti-rabbit IgG secondary antibody for 10 minutes at 37 °C. For visualization, streptavidin tablets were incubated with peroxidase at room temperature for 15 minutes, and then the streptavidin peroxidase was incubated with the slices for 15 minutes at 37°C, followed by DAB staining. The slices were subsequently stained with Meyer’s hematoxylin for 5 minutes. After dehydration, the slices were sealed with neutral glue. A score ranging from 0 to 4 was given based on the percentage of positive cell density:0, 0-3%, 1, 3-25%, 2, 26-50%, 3, 51-75%, and 4,76-100%. The staining intensity was also scored according to four levels: 0, no staining; 1, weak brown; 2, medium brown; and 3, strong brown. The final immune response score (IRS) was obtained as the product of the two scores and classified as follows: 0-1, negative expression; 2-3, mild positive expression; 4-7, moderate positive expression and 8-12, strongly positive expression (16). Based on the IRS, we divided the cervical cancer patients into the low CD155 expression group (IRS < 7) and high CD155 expression group (IRS > 7). The following antibodies were used: anti-CD155 (1:100, Cell Signaling Technology Danvers, MA, USA), anti-Ki67(1:300, Abcam), anti-p-AKT (1:100, Cell Signaling Technology Danvers, MA, USA) and p-pNF-κB65 (1:1000, Abcam).

### Immunofluorescence Staining

Cells were washed three times with phosphate-buffered saline (PBS), fixed with 4% paraformaldehyde for 15 minutes, and blocked with bovine serum albumin (BSA) at 37°C for 30 minutes. After washing three times with PBS, the cells were incubated with anti-LC3B (1:100, Cell Signaling Technology, Danvers, MA, USA), p-IKBα (1:200, GeneTex, USA) and p-NF-κBp65 (1:1000, Abcam), overnight at 4°C, followed by goat anti-rabbit antibody (1:200 Zhongshan Golden Bridge, Beijing, China) for 1 hour at 37°C in the dark. The cells were counterstained with DAPI (Yusen Biotech Inc, Shanghai, China) for 1 hour. Finally, the positive cells was analyzed by confocal fluorescence microscopy (Olympus Tokyo, Japan).

### Cell Lines and Cell Culture

CaSki and HeLa cell lines were from the Laboratory of Gynecological Oncology Center at Shandong University (Jinan, Shandong, China). CaSki and HeLa were cultured in RPMI 1640 or DMEM supplemented with 10% fetal bovine serum (all from Gibco, Grand Island, NY, USA), respectively, cultured in a humidified incubator at 37°C with 5% CO_2_.

### RNA Interference (RNAi)

SiRNAs were synthesized by Gene Pharma (Shanghai, China) with the following sequences: CD155 siRNA sequences 5-CCCGUAACGCCAUCAUCUUTT-3; antisense, 5-AAGAUGAUGGCGUUACGGGTT-3; AKT siRNA sequences 5-GCACUUUCGGCAAGGUGAUTT-3; antisense, 5-AUCACCUUGCCGAAAGUGC-TT-3; control NC-RNA sequences 5-UUCUCCGAACGUGUCACGUTT-3; antisense, 5-ACGUGACACGUUCGGAGAATT-3. Cells were transfected with siRNA CD155 or control siRNA when the cell confluence reached 50% using Lipofectamine 2000 reagent (Invitrogen, Carlsbad, California, USA) and Opti-MEM (Gibco, Grand Island, NY, USA) according to the manufacturers’ instructions.

### Construction and Transfection of the CD155 Overexpression Vector

The plasmid for overexpressing CD155 was purchased from OBIO Co., Ltd. (Shanghai, China). We used the lentiviral vector PGLVH1-Puro to improve the transfection efficiency. Forty-eight hours after lentiviral infection, puromycin (Amresco, Solon, OH, USA) was used to screen cell lines stably overexpressing CD155.

### ELISA Assay

The serum samples used to analyze CD155 levels were frozen at -80°C prior to performing ELISA. The ELISA kit was purchased from Biorbyt Company (Cambridge, UK) and used according to the manufacturer’s instructions.

### Western Blot

Cells were washed 3 times with PBS, and then lysed on ice in radioimmunoprecipitation analysis buffer (RIPA; Beyotime Institute of Biotechnology, China, 1% phenylmethylsulfonyl fluoride (PMSF); 1% NaF) for 30 minutes. The cell lysate was then centrifuged at 12,000 rpm for 10 minutes at 4°C. Next, the proteins were separated by SDS-PAGE and transferred to a PVDF membrane (Merck Millipore, Burlington, MA, USA). The membrane was incubated with primary antibody overnight, followed by incubation with the appropriate secondary antibody. Detection was realized using an enhanced chemiluminescence detection system.

### Cell Proliferation Assay

Cell viability was assessed using Cell Counting Kit-8 (CCK-8, Zhongshan Golden Bridge, Beijing, China). Cells (3x10^3^) were seeded in each well of a 96-well plate and cultured for r 0, 24, 48, 72, and 96 hours. After incubation with 10µl of CCK-8 for 2 hours, the absorbance at a 450nm was measured in a microplate reader (Infinite 2000; Tecan, Männedorf, Switzerland).

### Flow Cytometry

Cervical cancer cells were transfected with CD155 siRNA, PCMV CD155 or control. Approximately 48 hours later, the cells washed twice with pre-cooled PBS, and fixed in 75% alcohol at 4°C overnight. For cell cycle analysis, the cells were collected and stained with propidium iodide (PI) for 30 minutes in the dark (17, 18). To detect apoptosis, anapoptosis kit (62700-80, Biogems) was used. Cells were stained with 5μl of Annexin-V and 7AAD for 30 minutes in the dark at room temperature. The apoptotic ratio and cell cycle were quantitatively analyzed in a FACS Calibur flow cytometer (BD Biosciences, Franklin Lakes, NJ, USA). All results were analyzed with FlowJo v10.

### Transwell Migration and Invasion Assays

After digestion, the cells were suspended in 200 µl of serum-free 1640 or DMEM. The cells were then seeded into the upper chamber of a Transwell apparatus (pore size 8.0 µm; Costar, Cambridge, MA, USA) in the absence or presence of 100 µl of Matrigel Matrigel was diluted 1:8 in the serum-free medium (Corning, Corning, New York, USA). Medium with 20% FBS was added to the lower chamber as a chemoattractant. After 16 hours, the cells that passed through the filter were fixed with 4% paraformaldehyde (Beyotime, Beijing, China) for 5 minutes and stained with crystal violet (Beyotime, Beijing, China) (19). Images were captured with an Olympus IX51 inverted microscope (Olympus Tokyo, Japan). The number of migrated or invaded was counted cells in three random fields of view (magnification, 200x) of each chamber.

### Antibody Array Profiling of Cancer Signaling Phosphoproteins

The cancer signaling phosphoantibody microarray CSP100 plus designed and manufactured by Full Moon Biosystems, Inc. (Sunnyvale, California) was used. This microarray contains 304 antibodies. Each antibody has six replicates and multiple positive and negative controls and is printed on coated glass microscope slides. The antibody array experiment was performed by Wayen Biotechnology according to their established protocol (Shanghai, China).

### Immunoprecipitation

Collect the cells in the lysis buffer (Beyotime Biotechnology, China) and left on ice for 30min, sonicated, and centrifuged at 15000 rpm for 15 min at 4°C. Supernatants were collected. Each immunoprecipitation (IP) was performed with 10 μg antibody, 1000 μg protein, and incubated overnight at 4°C. The supernatant was incubated with protein A/G agarose beads (Santa Cruz, USA) for 6h. Wash the beads 3 times and boil, and immunoprecipitated proteins were detected using western blotting.

### Tumor Formation Assay in Nude Mice

Twelve specific pathogen-free (SPF)-grade female nude mice (18-22 g, 4-6 weeks old) were used in this study. The use of animals was approved by the Institute of Zoology, Shandong University. The mice were randomly allocated to the PCMV-CD155 group and NC-CD155 group, with six mice in each group. CaSki cells transfected with PCMV-CD155 or NC-CD155 were trypsinized, washed three times with PBS, and resuspended in PBS, and 200μl (1×107 cells) of the suspension was injected subcutaneously into the right armpit of each mouse. Tumor weight and volume and body weight of nude mice were measured every 2-3 days. The tumor volume is was calculated as 0.52×length×width^2^.

### Statistical Analysis

All experiments were independently repeated at least three times. GraphPad Prism 7.0 (GraphPad Software, La Jolla, CA, USA) was used for data processing and statistical analysis. The relationships between CD155 expression and clinicopathological parameters were analyzed using the χ2 test. For normally distributed data, the two-tailed Student’s t-test was used for statistical comparison between the two independent groups; a non-parametric test was used for, data that did not conform to a normal distribution. Results are reported as the mean ± standard deviation (SD). P< 0.05 was considered significant.

## Result

### CD155 Expression Is High in Cervical Cancer Tissues and Is Correlated With Differentiation and Ki67 Expression

**Figure 1:**
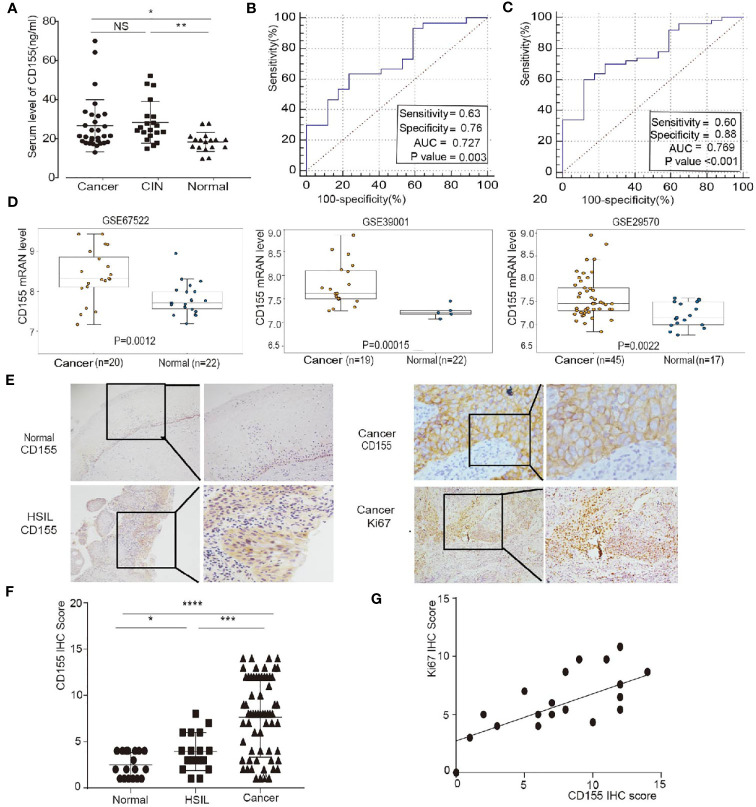
CD155 expression is up-regulated in patients with cervical cancer and HSIL and is positively correlated with Ki67 expression. **(A)** Distribution of serum CD155 levels CD155 in healthy women (n=17), HSIL patients (n=20) and cervical cancer patients (n=30). **(B, C)** Receiver operating characteristic (ROC) curve analysis of normal versus cervical cancer tissues and normal versus cervica cancer + HSIL tissues. The area under the ROC curve (AUC) was calculated for the diagnosis of cervical cancer. **(D)** Validation of CD155 expression in three GEO datasets: GSE67522, GSE39001 and GSE29570. CD155 mRNA levels were compared using Student’s t-test in the “limma” software package. **(E)** Representative images of CD155 immunohistochemistry (IHC) staining in normal cervical tissue (n = 18), HSIL tissue (n=20), and cervical cancer tissue (n=66). **(F)** Representative images of Ki67 IHC staining in cancer tissue (left: 100x, right: 200x). **(G)** CD155 expression was significantly positively correlated with the expression of Ki67 (r = 0.6608, P = 0.0004) in cervical cancers (data from IHC scores). NS, no significant. *P < 0.05, **P < 0.01, and ***P < 0.001.

The expression level of CD155 in sera from 15 healthy women, 20 HSIL patients, and 30 cervical cancer patients was determined by ELISA. CD155 expression was significantly higher in cervical cancer and HSIL patients (**Figure 1A**). We next evaluated the diagnostic significance of CD155 in cervical cancer and HSIL *via* receiver operating characteristic (ROC) analysis. CD155 had a high diagnostic value in cervical cancer patients, with an area under the curve (AUC) of 0.727, sensitivity of 0.63, and specificity of 0.76 (**Figure 1B**). In addition, for the diagnosis of HSIL + cancer AUC of CD155 was 0.769, with sensitivity of 0.6, and specificity of 0.88 (**Figure 1C**). The mRNA level of CD155 was analyzed using the “limma” software package and gene expression data for cervical cancer tissue and normal cervical tissue samples downloaded from the Gene Expression Omnibus (GEO). Consistent with the ELISA results, the transcription level of CD155 was higher in cervical cancer tissues than in normal cervix tissues (**Figure 1D**). To further examine the role of CD155 cervical cancer progression, the protein expression of CD155 in cervical cancer (n = 66), HSIL (n = 16), and normal cervix cervical (n = 18) was detected by IHC. The results showed that CD155 expression was localized in the cell membrane and cytoplasm. In addition, CD155 expression was significantly higher in cervical cancer tissues compared with HSIL and normal cervical tissues and in HSIL tissues compared with normal cervical tissues. Representative images are shown in (**Figures 1E, F**). The Analysis analysis of clinicopathological characteristics revealed a relationship between CD155 expression and low differentiation of cervical cancer (**Table 1**). Furthermore, Ki67 IHC staining of cervical cancer tissues (n=30), indicated that Ki67 expression in cervical cancer was positively correlated with CD155 expression (**Figure 1G**).

**Table 1:** Association between CD155 expression and the clinicopathological features of patients with cervical cancer.

Characteristics		CD155 expression		P
	Patient	Low	High	
	N = 65	N = 30	N = 35	
Age				
< 45	25	11	14	0.7997
≥45	40	16	24	
Clinicalstage				
Stage I	46	21	25	0.4082
Stage II/III	19	6	13	
Differentiation				
Low/moderat	53	19	34	0.0608
High	12	8	4	
Tumor size				
<4cm	40	17	23	>0.9999
≥4cm	25	10	15	
LNM				
Negative	44	20	24	0.4259
Positive	21	7	14	
DSI				
<1/2	15	10	5	0.0363*
≥1/2	50	17	33	

SCC, squamous cell carcinoma; LNM, lymph node metastasis; LVSI, lymph vascular space involvement; DSI, deep stromal invasion.

*Statistically significantly value.

### CD155 Regulates the Proliferation and Apoptosis of Cervical Cancer Cells

**Figure 2:**
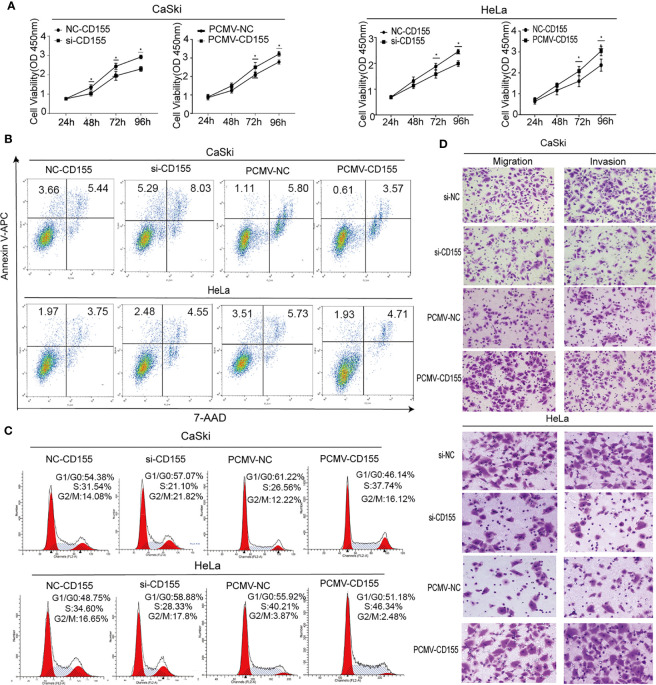
CD155 affects the biological behavior of cervical cancer cells. CaSki and HeLa cells were transfected with NC-CD155, si-CD155, PCMV-NC, and PCMV-CD155. **(A)** The viability of Hela and CaSki cells was measured by using the CCK8 assay. **(B)** Apoptosis was detected by flow cytometry after staining with Annexin V-APC and 7AAD. **(C)** Cell cycle analysis by flow cytometry. **(D)** Transwell assays were used to assess the invasion and migration capacity of CaSki and HeLa cells in which CD155 was overexpressed or knocked down. The data are the mean ± SEM of at least three independent experiments. *P < 0.05.

Silencing of CD155 expression or overexpression of CD155 was accomplished by transfection with siCD155 or CD155-PCMV in CaSki and HeLa cells. Compared with the control group, the efficiency of silencing or overexpression in CaSki and HeLa cells was greater than 60% at the protein level (**Supplementary Figure 1A**). The effect of CD155 expression on the proliferation of cervical cancer cells was detected by the CCK8 assay. Silencing CD155 significantly reduced the proliferation of HeLa and CaSki cells, whereas overexpression of CD155 significantly increased the proliferation of in both cell lines (**Figure 2A**). The association of CD155 associated with apoptosis. Forty-eight hours after transfection of CaSki or HeLa cells with siCD155, Annexin-V and 7AAD staining revealed obvious apoptosis compared with the NC group (**Figure 2B**), The apoptotic ratio in CaSki and HeLa cells was quantitatively analyzed with FlowJo v10 (**Supplementary Figure 2A**). Silencing CD155 increased the number of CaSki and HeLa cells in G0/G1 phase and reduced the number of cells in the S phase; the opposite effects were observed when CD155 was overexpressed in these cell lines. Compared with the control group, CaSki and HeLa cells transfected with siCD155 were blocked in G0/G1 phase (**Figure 2C**). Quantitative analysis of the cell cycle is shown in **Supplementary Figure 2B**. Finally, Transwell assays showed that cell migration and invasion were significantly reduced by silencing CD155 but significantly increased by overexpressing CD155 in CaSki and HeLa cells (**Figure 2D**). The results of the quantitative analysis are shown in **Supplementary Figures 2C, D**.

### The Effect of CD155 on the Expression of Proteins Related to the Cell Cycle and Apoptosis

**Figure 3:**
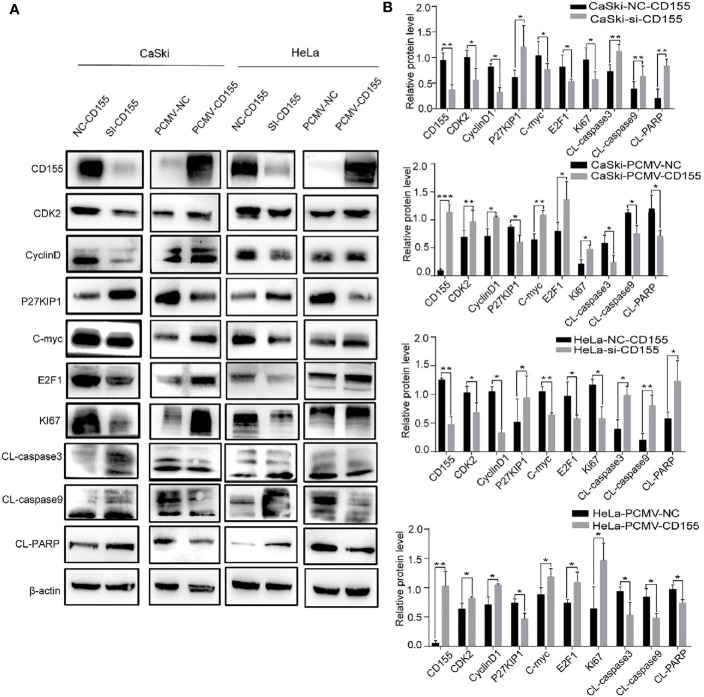
CD155 affects the expression of cell cycle- and apoptosis-related proteins in CaSki and HeLa cells. **(A)** After transfecting CaSki and HeLa cells with NC-CD155, si-CD155, PCMV-NC, or PCMV-CD155, the protein expression levels of CD155, CDK2, CyclinD1, P27KIP1, C-myc, E2F1, Ki67, CL-caspase3, CL-caspase9 and CL-PARP were analyzed by western blot. β-actin as a control. **(B)** Quantitation of the bands shown in **(A)** using Image J The data are the mean ± SEM of at least three independent experiments. *P < 0.05, **P < 0.01, and ***P < 0.001.

Proteins related to proliferation, apoptosis, and cyclin were analyzed by Western blot. Compared with the control group, silencing CD155 reduced the expression of the cell cycle-related proteins CDK2, CyclinD1, and C-myc, but increased P27KIP1 expression in HeLa and CaSki cells. Silencing CD155 also reduced the expression of the proliferation-related proteins E2F1and Ki67 while increasing the expression of the apoptosis-related proteins CL-PARP, CL-Caspase-3, and CL-Caspase-9. The same proteins were affected in the opposite manner by overexpressing CD155 in CaSki and HeLa cells (**Figure 3**).

### CD155 Regulates the AKT/mTOR Pathway, Autophagy, and the NF-κB Pathway in Cervical Cancer

**Figure 4:**
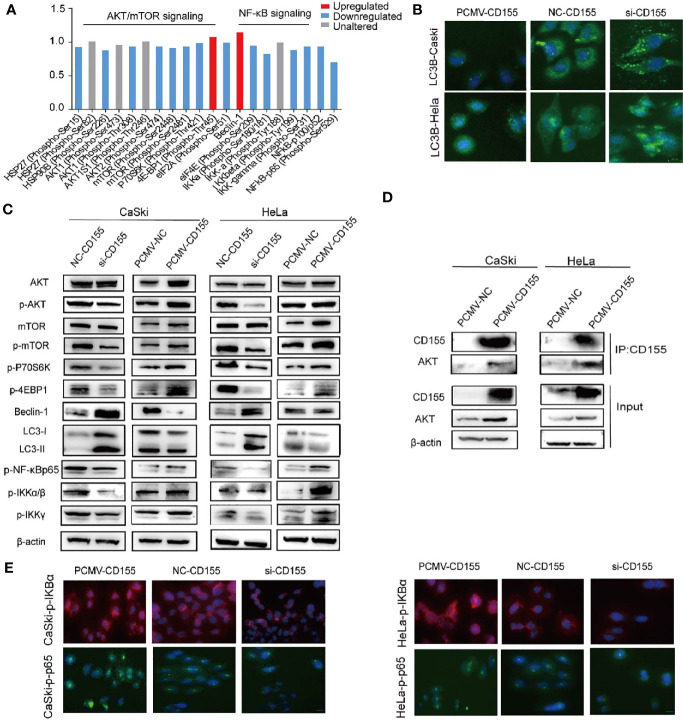
CD155 regulates the AKT/mTOR pathway, autophagy, and the NF-κB (NF-κB) pathway. **(A)** Phosphoproteome array analysis of phosphoprotein expression changes after CD155 knockdown in CaSki cells. Phosphoproteins whose levels increased or decreased by more than 12% are marked in red and blue, respectively. **(B)** Immunofluorescence staining of LC3B in CaSki and HeLa cells transfected with CD155 siRNA (si-CD155), negative control RNA (NC-CD155), or PCMV-CD155, Scale bar: 10 µm. **(C)** Western blot analysis of the protein levels of AKT, p-AKT, mTOR, p-mTOR, p-4EBP1, p-p70S6K, Beclin1, LC3I, LC3II, p-NF-κBp65, p-IKKα/β, and p-IKKγ in CaSki and HeLa cells transfected with NC-CD155, si-CD155, PCMV-NC, or PCMV-CD155. β-actin was used as a loading control. **(D)** The Co-IP results showed that CD155 interacted with AKT to form the CD155/AKT complex. The Co-IP and western blot results showed that in PCMV-CD155 CaSki and PCMV-CD155 HeLa cells, the abundance of the CD155/AKT complex was significantly increased. **(E)** Immunofluorescence for p-NF-κBp65, p-IKBα using CaSki and HeLa cells infected with CD155 siRNA (si-CD155), negative control RNA (NC-CD155), or PCMV-CD155, Scale bar: 10 µm. Data are the mean ± SEM of at least three independent experiments.

To explore the molecular mechanism of CD155 in cervical cancer progression, we used a phosphoprotein antibody array to screen more than 300 molecules in sixteen cancer-related pathways. Proteins related to the AKT/mTOR and NF-κB pathways showed the most obvious changes after CD155silencing (**Figure 4A**), and thus we focused on these pathways in our subsequent analyses Western blot analysis revealed that the LC3BII/LC3BI ratio and Beclin1 expression were increased in CD155- silenced CaSki and HeLa cells. By contrast, the LC3BII/LC3BI ratio and Beclin1 expression were significantly decreased in PCMV-CD155 CaSki and PCMV-CD155 HeLa cells (**Figure 4C**). Consistent with the Western blot results, immunofluorescence assay showed that the proportion of cells containing LC3B puncta (> 3) was increased in cells transfected with CD155 siRNA, but, decreased in cells transfected with PCMV-CD155 (**Figure 4B**). The above results indicate that CD155 knockdown significantly enhances autophagy in CaSki and HeLa cells, while overexpression of CD155 has the opposite effect. To determine whether CD155 regulates the AKT/mTOR pathway in cervical cancer cells, we examined the expression of AKT, p-AKT, mTOR, p-mTOR, p-4EBP1, and p-P70S6K expression, which are critical molecules in the AKT/mTOR pathway. Western blot results analysis showed that the total expression of AKT and mTOR was not significantly altered in CD155-silenced CaSki and HeLa cells, but p-AKT, p-mTOR, p-4EBP1, and p-P70S6K expression decreased significantly. By contrast, in PCMV-CD155 CaSki and PCMV-CD155 HeLa cells, the expression of p-AKT, p-mTOR, p-4EBP1, and p-P70S6K increased significantly. These results indicate that CD155 regulates the AKT/mTOR signaling pathway in cervical cancer cells and activates autophagy (**Figure 4C**).

We analyzed the expression of NF-κB pathway-related proteins in the cervical cancer cell lines. As shown in **Figure 4C**, silencing CD155 decreased the expression of NF-κB related proteins such as p-NF-κBP65, p-IKKα/β, and p-IKKγ whereas overexpression of CD155 had the opposite effect. The quantitative analysis of the expression of NF-κB-related proteins was presented in **Supplementary Figures S3**. The expression of p-IKBa and p-NF-κBp65 in CaSki and Hela cells were analyzed by the immunofluorescence method. CD155 overexpression promotes phosphorylation of IKBα, p-NF-κBP65 was activated and transferred to the nucleus and ultimately promoted cell proliferation and transcription. Silencing CD155 in CaSki and HeLa cells has opposite results (**Figure 4E**).

To further study whether there is a direct interaction between CD155 and AKT. We performed immunoprecipitation analysis. Co-IP analysis results showed that in CaSki and HeLa cells, CD155 can interact with AKT to form a CD155/AKT complex. To further explore the regulatory role of the CD155/AKT complex in CaSki and HeLa cells. The Co-IP and western blot results showed that, compared with that in CaSki and HeLa cells, the abundance of the CD155/AKT complex in PCMV-CD155 CaSki and PCMV-CD155 HeLa cells was significantly increased (**Figure 4D**).

### AKT Knockdown Reverses the Anti-Apoptotic and Inhibition of Autophagy and AKT/mTOR/NF-κB Pathway Activating Effects of CD155 Overexpression

**Figure 5:**
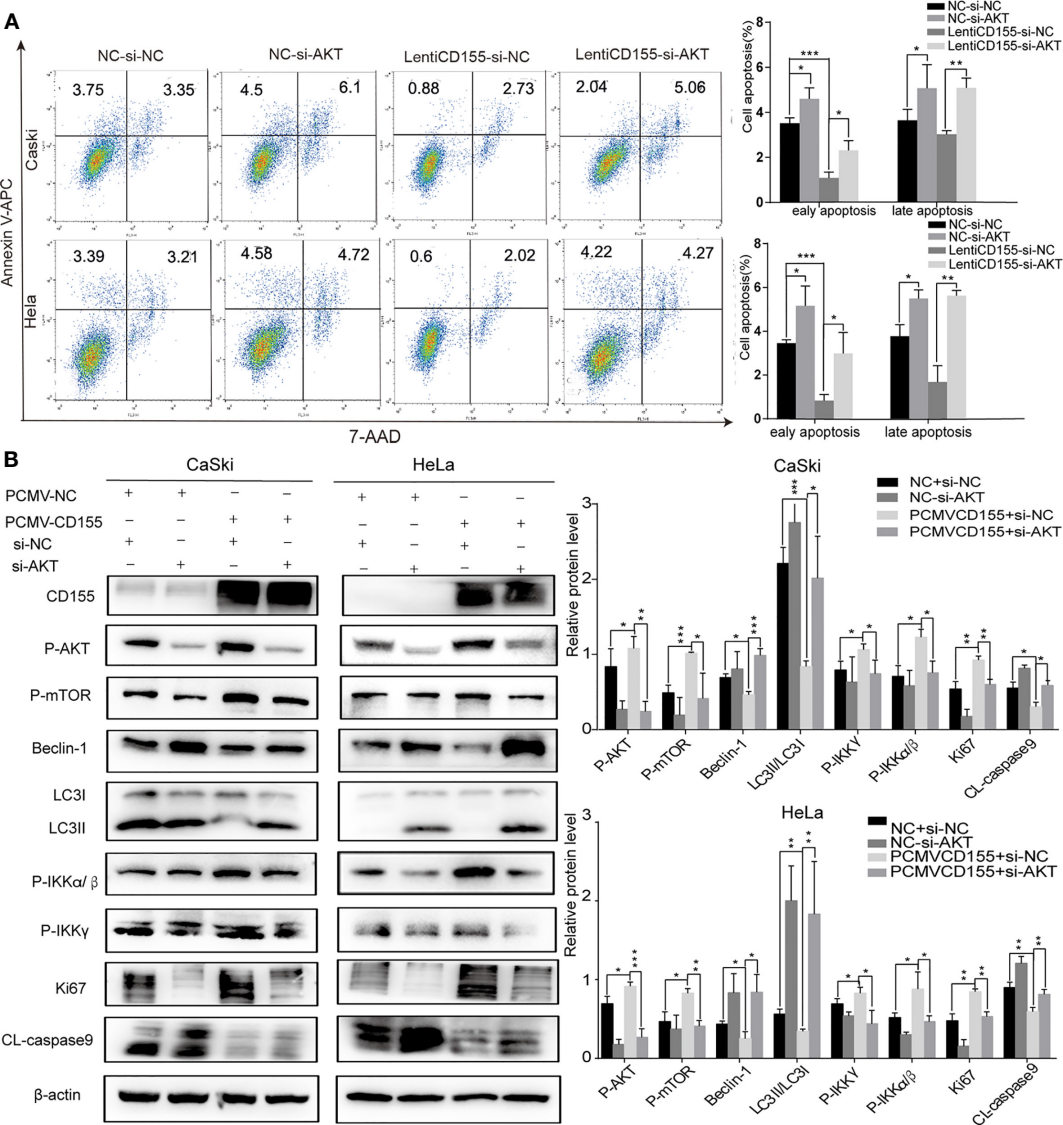
AKT knockdown reverses the anti- apoptotic effects and activation of the AKT/mTOR/NF-κB pathway induced by CD155 overexpression. CaSki and HeLa cells overexpressing CD155 were transfected with AKT-siRNA **(A)** Flow cytometry was used to detect apoptosis after staining with APC Annexin-V and 7AAD. The apoptotic ratio in CaSki and HeLa cells was quantitatively analyzed with FlowJo v10. **(B)** The expression of CD155, p-AKT, p-mTOR, Beclin-1, LC3BI, LCBII, p-IKKγ, p-IKKα/β, CL-caspase9, and Ki67 was measured by western blot. β-actin was used as a control. Quantitation was performed using Image J, and the data are the mean ± SEM of at least three independent experiments. *P < 0.05, **P < 0.01, and ***P < 0.001.

To examine whether the anti- apoptotic effect of CD155 is mediated by AKT/mTOR and AKT/NF-κB signaling pathway activation, siRNA targeting AKT was used to transfect cells overexpressing CD155. Forty-eight hours after transfection of siAKT, autophagy, cell apoptosis and cell proliferation were analyzed. Because our observations above indicated that p-AKT plays a role in the mechanism by which CD155 promotes cervical cancer progression, we detected the expression of p-AKT instead of AKT. Western-blot analysis showed nearly 70%. Subsequent flow cytometry and westernblot analyses showed that knockdown of AKT in CaSki and HeLa cells overexpressing CD155 reduced the anti-apoptotic effect of CD155 expression (**Figure 5A**). Knockdown of AKT significantly reduced the increases in p-mTOR, p-IKKα/β, and p-IKKγ expression induced by CD155 overexpression. At the same time, expression of the autophagy protein Beclin-1 and the LC3BII/LC3BI ratio increased significantly. Finally, knockdown of AKT reversed the increases in CL-caspase9 and Ki67 induced by overexpression of CD155 (**Figure 5B**).

### CD155 Overexpression Promotes Cervical Cancer Growth *In Vivo*

**Figure 6:**
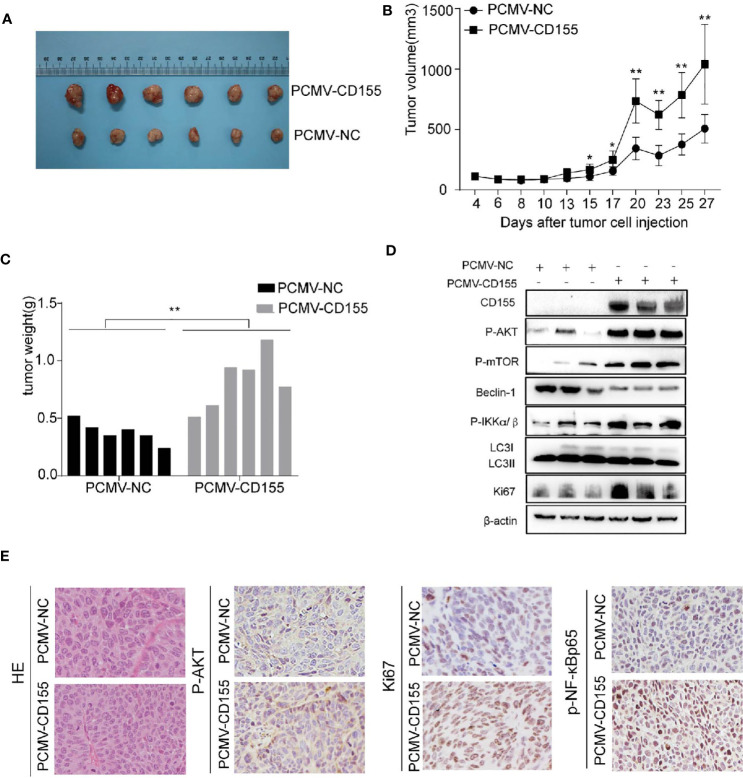
CD155 overexpression promotes cervical cancer growth *in vivo*. **(A)** Images of xenograft tumors derived from cells transfected with PCMV-CD155 or PCMV-NC. **(B)** The volumes of xenograft tumors derived from cells transfected with PCMV-CD155 or PCMV-NC. **(C)** The weight of xenograft the formed tumors derived from cells transfected with PCMV-CD155 or PCMV-NC. **(D)** Western blotting was used to detect protein expression in tumor forming tissues *in vivo*. The LC3BII/LC3BI ratio and protein expression of Beclin-1 were significantly decreased in the PCMV-CD155 group, while the protein expression of p-AKT, p-mTOR, P-IKKα/β and Ki67 was significantly increased. All data represent three independent experiments. The data were the mean ± SEM of at least three independent experiments. **(E)** Images of HE staining of xenograft tumors (200x). The expression of p-AKT, Ki67, and p-NF-κBp65 in xenograft tumors was detected by IHC (200x). *P < 0.05, **P < 0.01.

To further verify the effect of CD155 overexpression on the proliferation of cervical cancer cells *in vivo*, we constructed tumor xenograft models by injecting nude mice with CaSki cells transfected with PCMV-NC or PCMV-CD155. Tumor weight and volume were significantly higher in the PCMV-CD155 group than in the PCMV-NC group (**Figures 6A–C**). The expression of Ki67, p-AKT, and p-NF-κBP65 in xenograft mouse tissue was evaluated by IHC. Ki67, p-AKT, and p-NF-κBP65 staining was significantly stronger in tumor tissue from the PCMV-CD155 group than in tumor tissue from the control PCMV-NC group (**Figure 6E**). Western blotting was used to detect protein expression in tumor forming tissues *in vivo*. The LC3BII/LC3BI ratio and protein expression of Beclin-1 were significantly decreased in the PCMV-CD155 group, while the protein expression of p-AKT, p-mTOR, p-IKKα/β and Ki67 was significantly increased (**Figure 6D**). The above results indicate that the CD155 overexpression significantly promotes tumor growth *in vivo*.

## Discussion

CD155 is highly expressed in many tumors, and CD155 protein levels are closely related to tumor progression and poor prognosis (10, 11). Serum levels of soluble CD155 are significantly higher in cancer patients than in healthy volunteers and correlate positively with tumor staging (20). These studies support the potential of CD155 as a biomarker to assess cancer progression and prognosis, but the mechanism of CD155 in cervical cancer has not been examined. In the present study, we found that serum CD155 levels were elevated in cervical cancer and HSIL patients and also differed significantly between HSIL patients and healthy women. However, testing of larger samples is necessary to confirm the sensitivity and specificity of serum CD155 in detecting HSIL and cervical cancer patients and the efficiency and feasibility of cervical cancer screening combined with conventional HPV DNA testing and cytology need to be further evaluated and verified.

Ki67 is a marker of cell proliferation, initially defined by its city of origin (Kiel) and the number of original clones (21). Ki-67 is highly expressed in cycling cells but strongly down-regulated in resting G0 cells. Consequently, Ki67 is a clinically significant proliferation marker used to grade many types of cancer (22). Ki67 detection is widely used in the auxiliary diagnosis of cervical precancers and cancers (23). In the present study, analyses of clinical specimens and GEO datasets indicated that CD155 protein expression is up-regulated in cervical cancer. Furthermore, IHC analysis showed that CD155 expression is positively correlated with Ki67 expression. These results support a close relationship of CD155 with neoplasia and the development of cervical carcer.

CD155 has been previously shown to promote tumor growth. CD155 enhances serum-induced activation of Ras/Raf/MEK/ERK signaling by up-regulating cyclin D2 and E, down-regulating P27KIP1, and shortening the G0/G1 phase of the cell cycle in NIH3T3 cells, ultimately slowing cell proliferation (9). In addition, knocking out CD155 reduces tumor size and weight in colon cancer models and attenuates tumor metastasis rates in several other mouse tumor models (24, 25). In the present study flow cytometry, CCK8 cell proliferation assays and tumor xenotransplantation experiments showed that CD155 accelerates the formation and progression of cervical cancer by promoting the proliferation of cervical cancer cells through a shift from G0/G1 phase to S phase.

In the various stages of cell proliferation, diverse cell cycle proteins exhibit changes in expression and degradation patterns to coordinate mitotic events (26). In the present study, functional experiments showed that silencing CD155 inhibited cell proliferation by inducing cell cycle arrest at the transition from G0/G1 phase to S phase. The protein E2F ((E2 factor) is differentially expressed during the cell cycle and controls cell proliferation (27). E2F-1 is a crucial transcriptional regulator of the cell cycle transition between G1 and S phase (28). P27KIP1 is a cyclin-dependent kinase inhibitor (CKI) of G1 cyclin/CDK complexes (29). However, the pattern of binding of the P27KIP1 protein to cyclin D/CDK4,6 complexes is more complicated (30). Our results showed that silencing CD155 induces significant changes in cell cycle-related molecules, including down-regulation of CDK2, CyclinD1, E2F1, and C-myc, and up-regulation of P27KIP1. Overexpressing CD155 in CaSki and HeLa cells had the opposite effects on these proteins.

The expression of CD155 is closely related to the invasion and migration ability of tumor cells. Stimulation of CD155 with its ligand promotes the Src kinase-mediated phosphorylation of the ITIM of CD155, focal adhesion kinase (FAK), and Paxillin, ultimately inhibiting cell adhesion and improving cell viability (6). CD155, the PDGF receptor, and integrin αvβ3 form a ternary complex at the leading edge of the cell (31, 32) that plays a vital role in frontier dynamics and ultimately improves cell movement (4). Consistent with this role of CD155, our results showed that silencing CD155 reduces the invasion and migration ability of cervical cancer cells.

CD155 knockdown significantly induced apoptosis in colon cancer cells and increased the expression of CL-caspase-3 and CL-PARP. The apoptosis induced by CD155 knockdown seems to be related to an imbalance of anti-apoptotic and pro-apoptotic gene products (25). In the present study, the results of both flow cytometry analysis of apoptosis and western blot detection of the expression of apoptotic molecules in CD155-silenced cervical cancer cells were are consistent with these previous results.

Autophagy plays an important role in the occurrence, development and treatment of cancer. Although controversy remains, it is generally believed that autophagy inhibits cancer progression in the early stage of cancer but promotes progression in the middle and late stages (33, 34). The AKT/mTOR signaling pathway is a crucial pathway that regulates apoptosis, proliferation, and autophagy (35, 36). Here, we demonstrated for the first time that silencing CD155 promotes the autophagic flux of cervical cancer cells *via* regulation of the mTOR pathway. This study demonstrated that CD155 can interact with AKT and form CD155/AKT complex in Caski and HeLa cells through CO-IP assay. We further found that the CD155/AKT complex expression was increased in PCMV-CD155 CaSki and PCMV-CD155 HeLa cells. Furthermore, we showed that CD155 regulates the mTOR pathway by promoting the phosphorylation of AKT. In this study, silencing CD155 in cervical cancer cells significantly reduced p-AKT, p-mTOR, p-P7S60, and p-4EBP1 expression levels and cell proliferation whereas autophagy and apoptosis were significantly increased compared with control cells. The opposite pattern was observed in cervical cancer cells overexpressing CD155.

The NF-κB pathway is related to tumor cell adhesion, angiogenesis, inflammation, and metastasis (37), and blocking NF-κB activity increases apoptosis (38). In addition, NF-κB is involved in the regulation of the cell surface expression of adhesion molecules such as E-selectin, vascular cell adhesion molecule-1, and intercellular adhesion molecule-1 (39). Consistent with these effects, the flavone morusin inhibits human cervical cancer growth and migration through NF-κB attenuation (40). Here, we found that CD155 regulates the proliferation and apoptosis of cervical cancer cells *via* the regulation of NF-κB. Western blot analysis revealed that silencing CD155 can down-regulates p-NF-κBp65, p-IKKα/β, and p-IKKγ, whereas overexpression of CD155 has the opposite effect.

**Figure 7:**
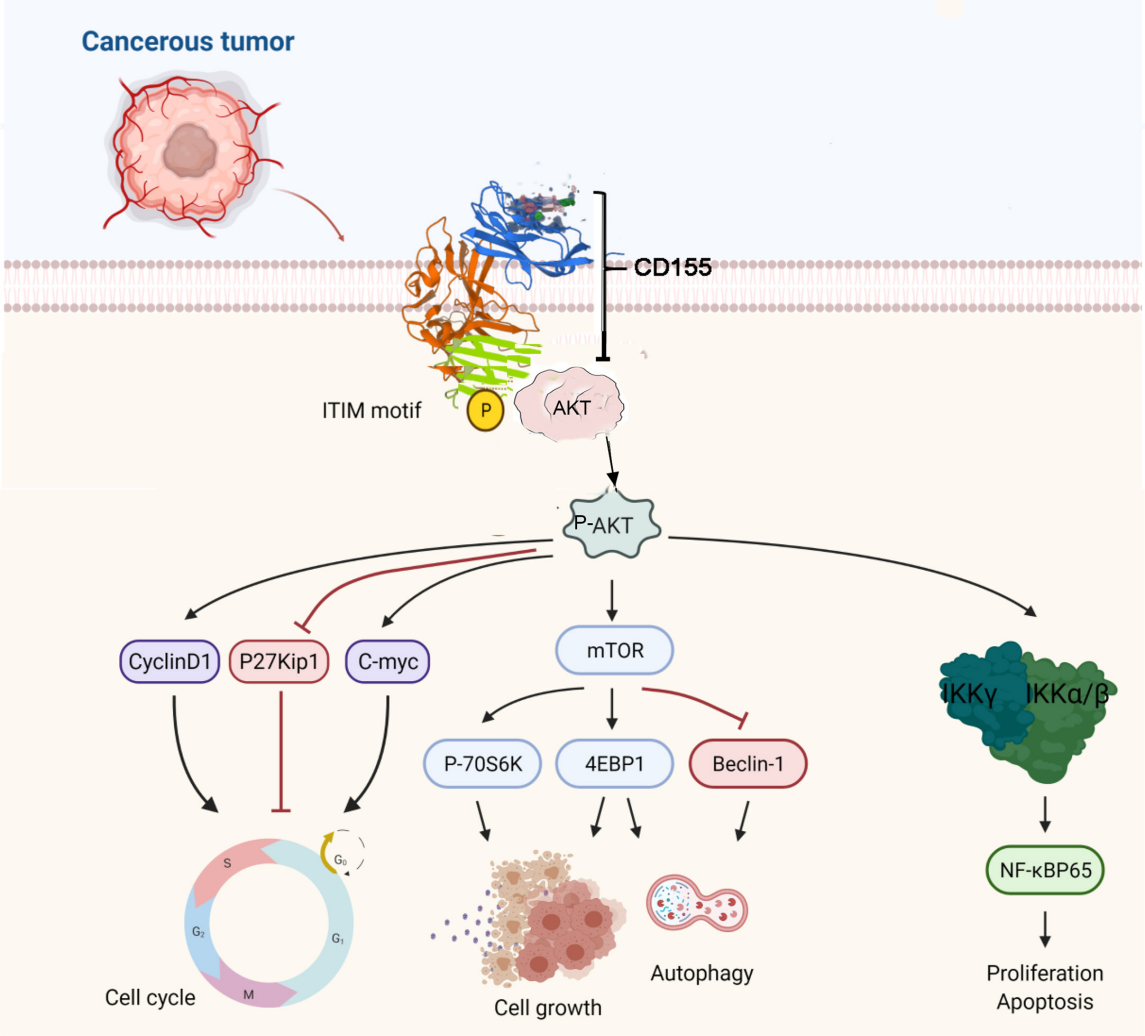
The proposed mechanism of CD155 in cervical cancer cells.

We further verify the mechanism between CD155 and AKT through rescue experiments. AKT knockdown confirmed that the anti-apoptotic and inhibition of autophagy effects of CD155 overexpression are mediated by activation of the AKT/mTOR and NF-κB signaling pathways. Transfection of CaSki and HeLa cells stably overexpressing CD155 with si-AKT increased the rate of apoptosis and autophagy. In addition, it reduced the increases in the expression of AKT/mTOR and NF-κB related proteins induced by CD155 overexpression. These findings suggest that CD155 Promotes the Progression of Cervical Cancer Cells through AKT/mTOR and NF-kB Pathways (**Figure 7**).

The effects of CD155 on cervical cancer progression involve mechanisms other than AKT/mTOR/NF-κB. Furthermore, CD155 also plays an immunosuppressive role in tumor progression, which was not examined here. Addressing these limitations will be the focus of our subsequent work.

## Conclusion

Our research demonstrated that CD155 can interact with AKT to form a complex, activates the AKT/mTOR/NF-κB pathway and inhibit autophagy and apoptosis. In addition, CD155 promotes cell cycle progression by up-regulating cyclinD1 and down-regulating the expression of P27KIP1. These findings suggest that CD155 may be a valuable target for the treatment of cervical cancer.

## Data Availability Statement

The original contributions presented in the study are included in the article/**Supplementary Material**, further inquiries can be directed to the corresponding author.

## Ethics Statement

The studies involving human participants were reviewed and approved by Ethics Committee of Shandong University. The patients/participants provided their written informed consent to participate in this study. The animal study was reviewed and approved by Institute of Zoology, Shandong University.

## Author Contributions

YZ and LL contributed to the conception and design of this study. YW and AW contributed to data acquisition. XY, SH and YS contributed to data curation. CG, WL and JZ contributed to the methodology and visualization. All authors contributed to manuscript review. All authors contributed to the article and approved the submitted version.

## Funding

The study was conducted at Qilu Hospital, Shandong University and was supported by the National Natural Science Foundation of China (NSFC, 81902644). Shandong Provincial Natural Science Foundation, China (ZR2019BC059 and ZR2020QH248). Science and Technology Development Project of Shandong Province (2019GSF108126) and the Key Research Project of Shandong Province (2017CXGC1210). Weifang Health Fund (2018–053).

## Conflict of Interest

The authors declare that the research was conducted in the absence of any commercial or financial relationships that could be construed as a potential conflict of interest.

## Supplementary Material

Supplementary Figure 1 CaSki and HeLa cells were transfected with NC-CD155, si-CD155, PCMV-NC. or PCMV-CD155. **(A, B)** Protein levels of CD155 and β-actin in CaSki or HeLa cells were analyzed by western blot. (mean ± SEM, *p < 0.05, **p < 0.01, ***p < 0.001).

Supplementary Figure 2 CaSki and HeLa cells were transfected with NC-CD155, si-CD155, PMC-NC, or PMV-CD155. **(A)** After Annexin-V and 7AAD staining, cell apoptosis was detected by flow cytometry. The apoptotic ratio in CaSki and HeLa cells was quantitatively with FlowJo v10. **(B)** Cell cycle analysis by flow cytometry and quantitative analysis of the cell cycle a were shown **(C, D)** Quantitative analysis of the invasion and migration of CaSki and HeLa cells in which CD155 was knocked or overexpressed. (mean ± SEM, *p < 0.05, **p < 0.01).

Supplementary Figure 3 CaSki and HeLa cells were transfected with NC-CD155, si-CD155, PMC-NC, or PMV-CD155. (A, B) The proportions of CaSki and HeLa cells containing LC3B puncta (> 5). **(C–F)** Quantification of relative protein expression levels in **Figure 4C**, **D**. (mean ± SEM; *p < 0.05, **p < 0.01).

Supplementary Figure 4
**(A)** The levels of related proteins in in tumorforming tissues *in vivo*. β-actin was used as a control. Quantitation was performed of western-blot assay using Image J. The data were the mean ± SEM. **(B)** Comparison of p-AKT expression in xenograft tumors. **(C)** Comparison of Ki67 expression in xenograft tumors. **(D)** Comparison of p-NF-κBp65 expression in xenograft tumors. *P < 0.05, **P < 0.01, and ***P < 0.001.

## References

[1] Cheah P-L Looi L-M Teoh K-H Mun K-S Nazarina AR. p16(INK4a) is a Useful Marker of Human Papillomavirus Integration Allowing Risk Stratification for Cervical Malignancies. Asian Pac J Cancer Prev (2012) 13(2):469–72. 10.7314/APJCP.2012.13.2.469 22524808

[2] zur Hausen H. Papillomaviruses and Cancer: From Basic Studies to Clinical Application. Nat Rev Cancer (2002) 2(5):342–50. 10.1038/nrc798 12044010

[3] Bhat S Kabekkodu S Noronha A Satyamoorthy K. Biological Implications and Therapeutic Significance of DNA Methylation Regulated Genes in Cervical Cancer. Biochimie (2016) 121:298–311. 10.1016/j.biochi.2015.12.018 26743075

[4] Takai Y Miyoshi J Ikeda W Ogita H. Nectins and Nectin-Like Molecules: Roles in Contact Inhibition of Cell Movement and Proliferation. Nat Rev Mol Cell Biol (2008) 9(8):603–15. 10.1038/nrm2457 18648374

[5] Koike S Horie H Ise I Okitsu A Yoshida M Iizuka N. The Poliovirus Receptor Protein is Produced Both as Membrane-Bound and Secreted Forms. EMBO J (1990) 9(10):3217–24. 10.1002/j.1460-2075.1990.tb07520.x PMC552052 2170108

[6] Oda T Ohka S Nomoto A. Ligand Stimulation of CD155alpha Inhibits Cell Adhesion and Enhances Cell Migration in Fibroblasts. Biochem Biophys Res Commun (2004) 319(4):1253–64. 10.1016/j.bbrc.2004.05.111 15194502

[7] Lange R Peng X Wimmer E Lipp M Bernhardt G. The Poliovirus Receptor CD155 Mediates Cell-to-Matrix Contacts by Specifically Binding to Vitronectin. Virology (2001) 285(2):218–27. 10.1006/viro.2001.0943 11437656

[8] Reymond N Imbert A Devilard E Fabre S Chabannon C Xerri L. DNAM-1 and PVR Regulate Monocyte Migration Through Endothelial Junctions. J Exp Med (2004) 199(10):1331–41. 10.1084/jem.20032206 PMC2211807 15136589

[9] Kakunaga S Ikeda W Shingai T Fujito T Yamada A Minami Y. Enhancement of Serum- and Platelet-Derived Growth Factor-Induced Cell Proliferation by Necl-5/Tage4/poliovirus Receptor/CD155 Through the Ras-Raf-MEK-ERK Signaling. J Biol Chem (2004) 279(35):36419–25. 10.1074/jbc.M406340200 15213219

[10] Nakai R Maniwa Y Tanaka Y Nishio W Yoshimura M Okita Y. Overexpression of Necl-5 Correlates With Unfavorable Prognosis in Patients With Lung Adenocarcinoma. Cancer Sci (2010) 101(5):1326–30. 10.1111/j.1349-7006.2010.01530.x PMC11158505 20331633

[11] Nishiwada S Sho M Yasuda S Shimada K Yamato I Akahori T. Clinical Significance of CD155 Expression in Human Pancreatic Cancer. Anticancer Res (2015) 35(4):2287–97. 25862891

[12] Carlsten M Norell H Bryceson Y Poschke I Schedvins K Ljunggren H. Primary Human Tumor Cells Expressing CD155 Impair Tumor Targeting by Down-Regulating DNAM-1 on NK Cells. J Immunol (Baltimore Md 1950) (2009) 183(8):4921–30. 10.4049/jimmunol.0901226 19801517

[13] Pende D Spaggiari G Marcenaro S Martini S Rivera P Capobianco A. Analysis of the Receptor-Ligand Interactions in the Natural Killer-Mediated Lysis of Freshly Isolated Myeloid or Lymphoblastic Leukemias: Evidence for the Involvement of the Poliovirus Receptor (CD155) and Nectin-2 (Cd112). Blood (2005) 105(5):2066–73. 10.1182/blood-2004-09-3548 15536144

[14] Gromeier M Lachmann S Rosenfeld M Gutin P Wimmer E. Intergeneric Poliovirus Recombinants for the Treatment of Malignant Glioma. Proc Natl Acad Sci USA (2000) 97(12):6803–8. 10.1073/pnas.97.12.6803 PMC18745 10841575

[15] Huang D Huang M Lin X Huang Q. CD155 Expression and its Correlation With Clinicopathologic Characteristics, Angiogenesis, and Prognosis in Human Cholangiocarcinoma. Onco Targets Ther (2017) 10:3817–25. 10.2147/ott.S141476 PMC5546808 28814880

[16] Fedchenko N Reifenrath J. Different Approaches for Interpretation and Reporting of Immunohistochemistry Analysis Results in the Bone Tissue - a Review. Diagn Pathol (2014) 9:221. 10.1186/s13000-014-0221-9 25432701 PMC4260254

[17] Zhang F Zhang Y Sun Y Ma R Thakur K Zhang J. Asparagus Officinalisasparanin A From L. Induces G0/G1 Cell Cycle Arrest and Apoptosis in Human Endometrial Carcinoma Ishikawa Cells Via Mitochondrial and PI3K/AKT Signaling Pathways. J Agric Food Chem (2020) 68(1):213–24. 10.1021/acs.jafc.9b07103 31861958

[18] Sun Y Thakur K Hu F Zhang J Wei Z. Icariside II Inhibits Tumorigenesis Via Inhibiting AKT/Cyclin E/ CDK 2 Pathway and Activating Mitochondria-Dependent Pathway. Pharmacol Res (2020) 152:104616. 10.1016/j.phrs.2019.104616 31883767

[19] Sun Y Thakur K Hu F Cespedes-Acuña C Zhang J Wei Z. Icariside II Suppresses Cervical Cancer Cell Migration Through JNK Modulated Matrix metalloproteinase-2/9 Inhibition *In Vitro* and *In Vivo* . Biomed Pharmacother Biomed Pharmacother (2020) 125:110013. 10.1016/j.biopha.2020.110013 32092821

[20] Yoshida J Ishikawa T Doi T Ota T Yasuda T Okayama T. Clinical Significance of Soluble Forms of Immune Checkpoint Molecules in Advanced Esophageal Cancer. Med Oncol (Northwood London England) (2019) 36(7):60. 10.1007/s12032-019-1285-x 31134385

[21] Menon S Guruvayoorappan C Sakthivel K Rasmi R. Ki-67 Protein as a Tumour Proliferation Marker. Clin Chim Acta; Int J Clin Chem (2019) 491:39–45. 10.1016/j.cca.2019.01.011 30653951

[22] Dowsett M Nielsen T A’Hern R Bartlett J Coombes R Cuzick J. Assessment of Ki67 in Breast Cancer: Recommendations From the International Ki67 in Breast Cancer Working Group. J Natl Cancer Institute (2011) 103(22):1656–64. 10.1093/jnci/djr393 PMC3216967 21960707

[23] Yu L Wang L Zhong J Chen S. Diagnostic Value of P16ink4a, Ki-67, and Human Papillomavirus L1 Capsid Protein Immunochemical Staining on Cell Blocks From Residual Liquid-Based Gynecologic Cytology Specimens. Cancer Cytopathol (2010) 118(1):47–55. 10.1002/cncy.20061 20069634

[24] Li X Das I Lepletier A Addala V Bald T Stannard K. CD155 Loss Enhances Tumor Suppression Via Combined Host and Tumor-Intrinsic Mechanisms. J Clin Invest (2018) 128(6):2613–25. 10.1172/jci98769 PMC5983325 29757192

[25] Zheng Q Wang B Gao J Xin N Wang W Song X. CD155 Knockdown Promotes Apoptosis Via AKT/Bcl-2/Bax in Colon Cancer Cells. J Cell Mol Med (2018) 22(1):131–40. 10.1111/jcmm.13301 PMC5742678 28816021

[26] Schafer K. The Cell Cycle: A Review. Veterinary Pathol (1998) 35(6):461–78. 10.1177/030098589803500601 9823588

[27] Bertoli C Skotheim JM de Bruin RA. Control of Cell Cycle Transcription During G1 and S Phases. Nat Rev Mol Cell Biol (2013) 14(8):518–28. 10.1038/nrm3629 PMC4569015 23877564

[28] Tsantoulis P Gorgoulis V. Involvement of E2F Transcription Factor Family in Cancer. Eur J Cancer (Oxford Engl 1990) (2005) 41(16):2403–14. 10.1016/j.ejca.2005.08.005 16213134

[29] Lee J Kim S. The Function of P27 KIP1 During Tumor Development. Exp Mol Med (2009) 41(11):765–71. 10.3858/emm.2009.41.11.102 PMC2788730 19887899

[30] Besson A Dowdy S Roberts J. CDK Inhibitors: Cell Cycle Regulators and Beyond. Dev Cell (2008) 14(2):159–69. 10.1016/j.devcel.2008.01.013 18267085

[31] Minami Y Ikeda W Kajita M Fujito T Amano H Tamaru Y. Necl-5/poliovirus Receptor Interacts in Cis With Integrin alphaVbeta3 and Regulates its Clustering and Focal Complex Formation. J Biol Chem (2007) 282(25):18481–96. 10.1074/jbc.M611330200 17446174

[32] Takahashi M Rikitake Y Nagamatsu Y Hara T Ikeda W Hirata K. Sequential Activation of Rap1 and Rac1 Small G Proteins by PDGF Locally at Leading Edges of NIH3T3 Cells. Genes Cells (2008) 13(6):549–69. 10.1111/j.1365-2443.2008.01187.x 18422604

[33] Amaravadi R Kimmelman AC White E. Recent Insights Into the Function of Autophagy in Cancer. Genes Dev (2016) 30(17):1913–30. 10.1101/gad.287524.116 PMC5066235 27664235

[34] White E. Deconvoluting the Context-Dependent Role for Autophagy in Cancer. Nat Rev Cancer (2012) 12(6):401–10. 10.1038/nrc3262 PMC3664381 22534666

[35] Kim YC Guan KL. mTOR: A Pharmacologic Target for Autophagy Regulation. J Clin Invest (2015) 125(1):25–32. 10.1172/JCI73939 25654547 PMC4382265

[36] Martini M De Santis M Braccini L Gulluni F Hirsch E. PI3K/AKT Signaling Pathway and Cancer: An Updated Review. Ann Med (2014) 46(6):372–83. 10.3109/07853890.2014.912836 24897931

[37] Ravi R Bedi A. NF-Kappab in Cancer–a Friend Turned Foe. Drug Resistance Updates Rev Commentaries Antimicrobial Anticancer Chemother (2004) 7(1):53–67. 10.1016/j.drup.2004.01.003 15072771

[38] Wang C Mayo M Baldwin A. TNF- and Cancer Therapy-Induced Apoptosis: Potentiation by Inhibition of NF-Kappab. Science (1996) 274(5288):784–7. 10.1126/science.274.5288.784 8864119

[39] Read M Neish A Luscinskas F Palombella V Maniatis T Collins T. The Proteasome Pathway is Required for Cytokine-Induced Endothelial-Leukocyte Adhesion Molecule Expression. Immunity (1995) 2(5):493–506. 10.1016/1074-7613(95)90030-6 7538441

[40] Wang L Guo H Yang L Dong L Lin C Zhang J. Morusin Inhibits Human Cervical Cancer Stem Cell Growth and Migration Through Attenuation of NF-κb Activity and Apoptosis Induction. Mol Cell Biochem (2013) 379:7–18. 10.1007/s11010-013-1621-y 23543150

