# Supplementary figures and images for: CD155 Promotes the Progression of Cervical Cancer Cells Through AKT/mTOR and NF-κB Pathways
Source: Front Oncol. 2021 Jun 7;11:655302. doi: 10.3389/fonc.2021.655302 (PMC8216081; doi:10.3389/fonc.2021.655302)

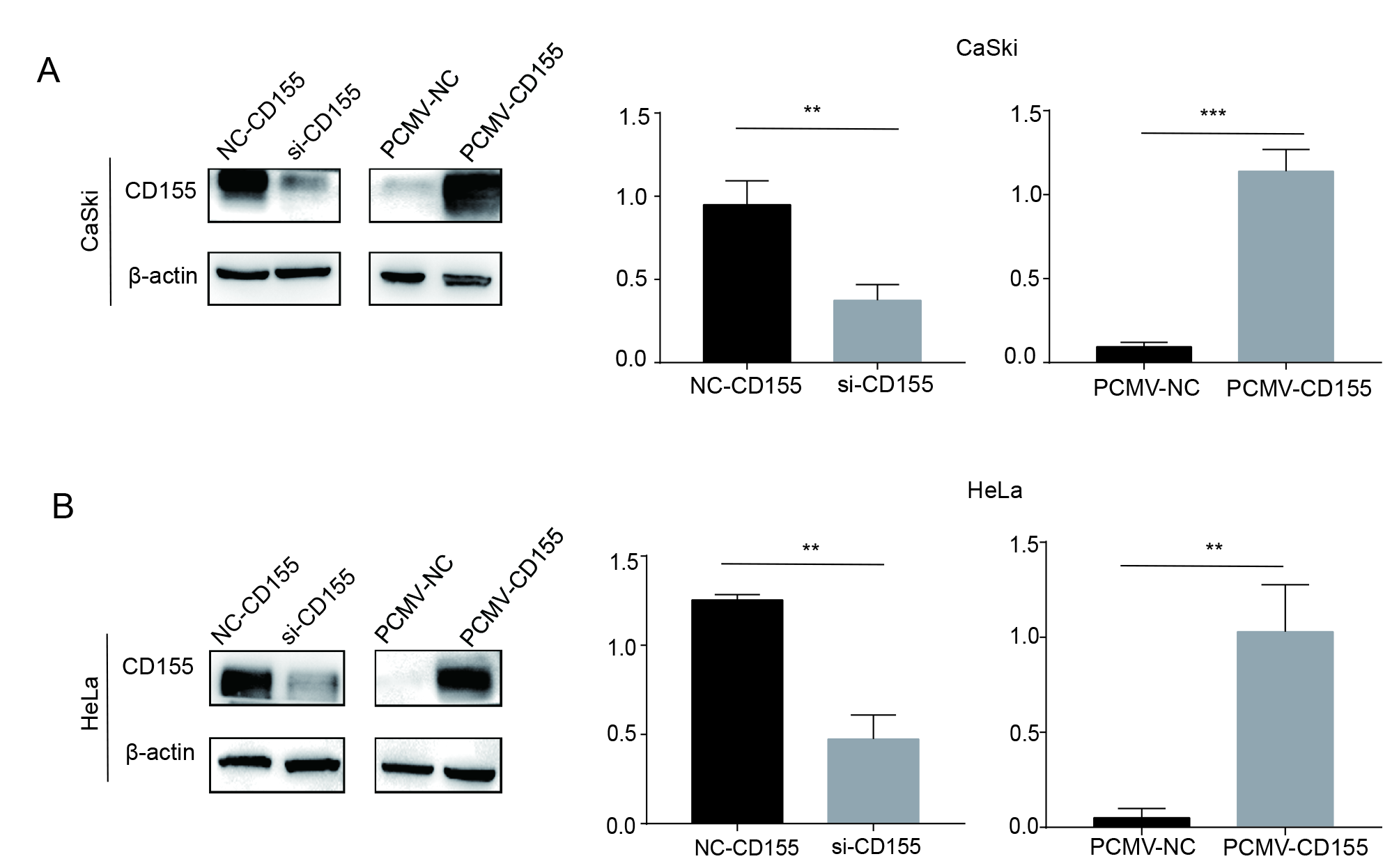

Supplement: Supplementary Figure 1 — CaSki and HeLa cells were transfected with NC-CD155, si-CD155, PCMV-NC. or PCMV-CD155. (A, B) Protein levels of CD155 and β-actin in CaSki or HeLa cells were analyzed by western blot. (mean ± SEM, *p < 0.05, **p < 0.01, ***p < 0.001). [file Image_1.tif]

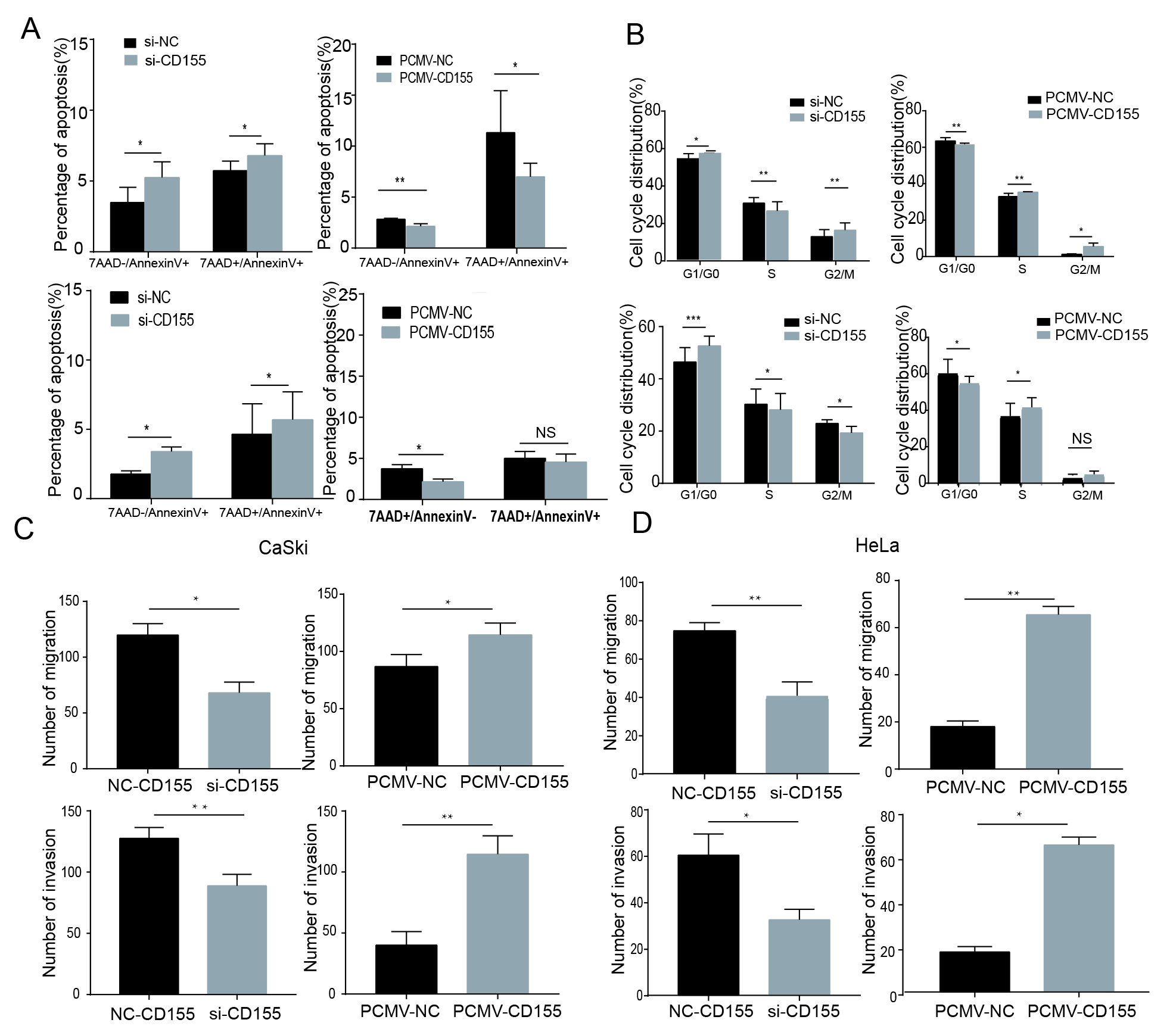

Supplement: Supplementary Figure 2 — CaSki and HeLa cells were transfected with NC-CD155, si-CD155, PMC-NC, or PMV-CD155. (A) After Annexin-V and 7AAD staining, cell apoptosis was detected by flow cytometry. The apoptotic ratio in CaSki and HeLa cells was quantitatively with FlowJo v10. (B) Cell cycle analysis by flow cytometry and quantitative analysis of the cell cycle a were shown (C, D) Quantitative analysis of the invasion and migration of CaSki and HeLa cells in which CD155 was knocked or overexpressed. (mean ± SEM, *p < 0.05, **p < 0.01). [file Image_2.tif]

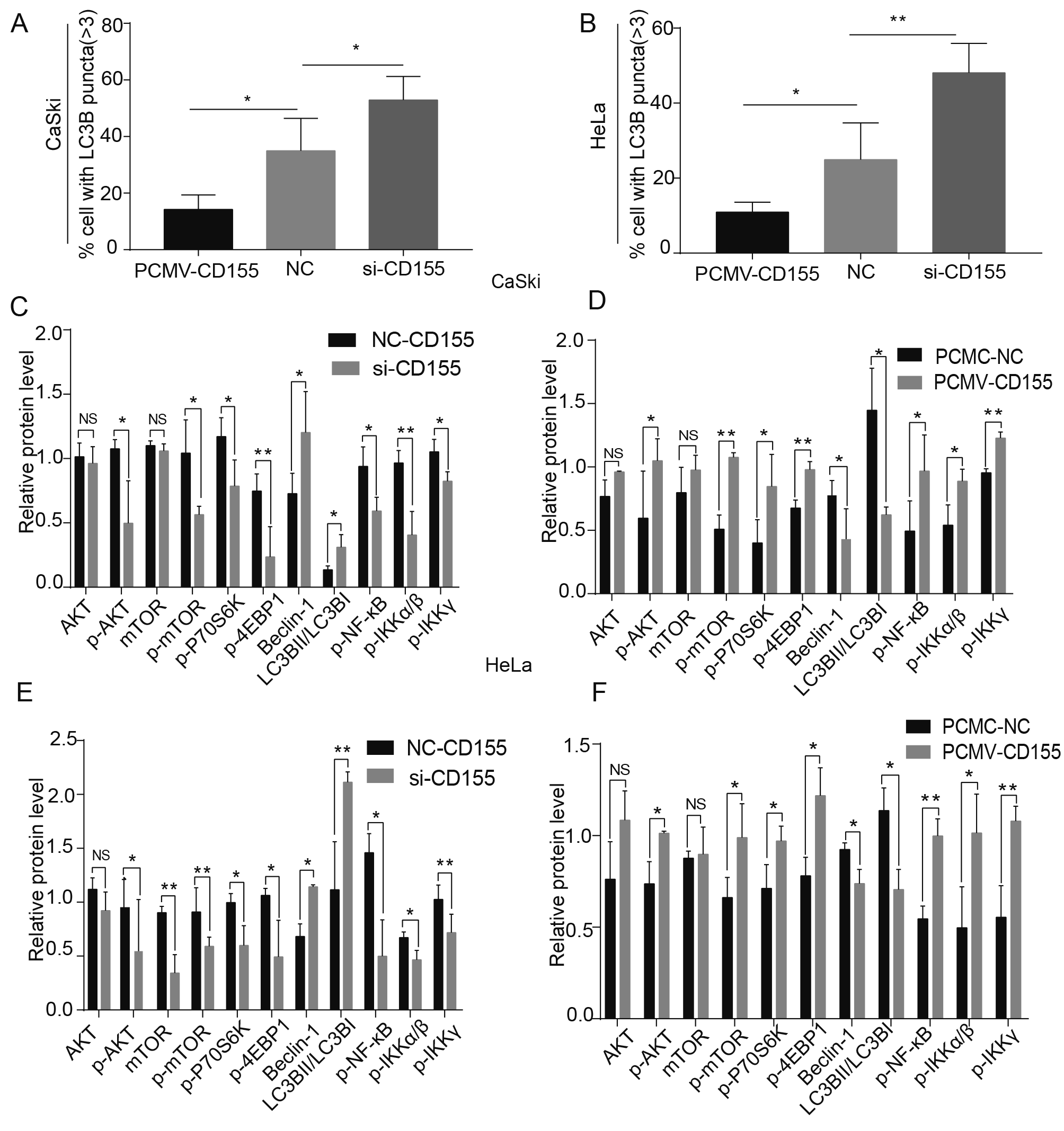

Supplement: Supplementary Figure 3 — CaSki and HeLa cells were transfected with NC-CD155, si-CD155, PMC-NC, or PMV-CD155. (A, B) The proportions of CaSki and HeLa cells containing LC3B puncta (> 5). (C–F) Quantification of relative protein expression levels in Figure 4C , D . (mean ± SEM; *p < 0.05, **p < 0.01). [file Image_3.tif]

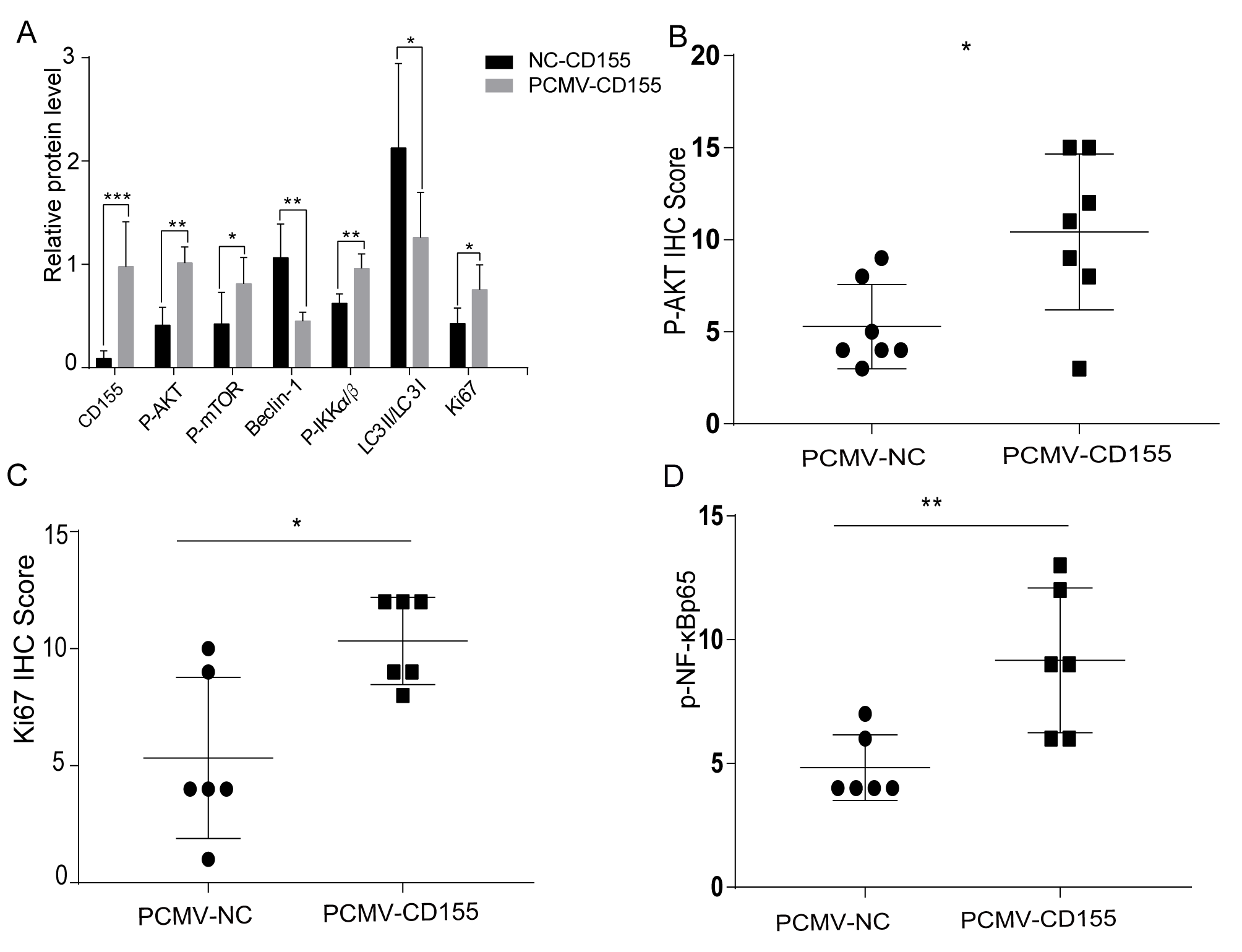

Supplement: Supplementary Figure 4 — (A) The levels of related proteins in in tumorforming tissues in vivo. β-actin was used as a control. Quantitation was performed of western-blot assay using Image J. The data were the mean ± SEM. (B) Comparison of p-AKT expression in xenograft tumors. (C) Comparison of Ki67 expression in xenograft tumors. (D) Comparison of p-NF-κBp65 expression in xenograft tumors. *P < 0.05, **P < 0.01, and ***P < 0.001. [file Image_4.tif]
